# The Brain-Worker’s Doom

**Published:** 1872-09

**Authors:** 


### THE BRAIN-WORKER'S DOOM.

WHENEVER a thinker, or student, or scholar gets into that condition when he feels very tired all over, at the close of the day, especially tired in the legs from the knees downwards, he ought to take heed to kindly nature's warning; the brain is giving out; not that he is going crazy, but he is getting into that nervous condition which makes instant abandonment of all mental application most imperatively necessary; without such prompt action, the whole machinery of the nervous system may become disordered, and months and years may not suffice to repair the damage; it means that the nervous energy is so nearly exhausted that there is not vitality to send it to the extremities; these energies have got such a set toward the brain that their consumption is in that direction; the magnet is there, drawing all into itself. It is just at this point that the

### MOVEMENT CURE

is most rational, most applicable, and most efficient, physical motion of the limbs, and little or no action of the brain, so as to change the current of nervous flow and set it in another direction, to parts which most need it, and thus re-establish the equilibrium. It is wonderful to note the change which a single day's excursion to the country will make, especially if several hours are spent in walking or on horseback. But if a man has been suffering with weak legs for months, he cannot expect so prompt a change in his feelings.

If overworked, over-anxious wives have these feelings from the responsibilities of household cares, the generous husband will hie them to the country right away, or if in the country already, will bring them to the city, that, seeing its sights and walking its streets, the ruts of the nervous currents may be changed, and the necessary repairs made, before sleepless nights come on, or the lightning stroke of the palsy, or the sundering of the heart-strings, close the history.

